# Correction: The MARC SE-Africa dashboard: Joining forces to counteract emerging antimalarial resistance in South and East Africa

**DOI:** 10.1371/journal.pdig.0001596

**Published:** 2026-07-20

**Authors:** Stephanie van Wyk, Ishen Seocharan, Eulambius M. Mlugu, Dhol S. Ayuen, Donnie Mategula, Tikhala Makhaza, James Kiarie, Victor Asua, Jimmy Opigo, Aimable Mbituyumuremyi, Kibor Kipkemoi Keitany, Emmah Mongina Nyandigisi, Pierre Sinarinzi, Peter Aguek Kon Baak, Tommy Nseka Manbul, Samwel Lazaro Nhiga, Sijenunu Aron Mwaikambo, Maulid Kassim, Sija Joseph Sija, Abdikarin Hussein Hassan, Michael Katende, Jaishree Raman, Karen I. Barnes

In Fig 5, the y-axis is incorrect. Please see the correct Fig 5 here.

**Fig 5:**
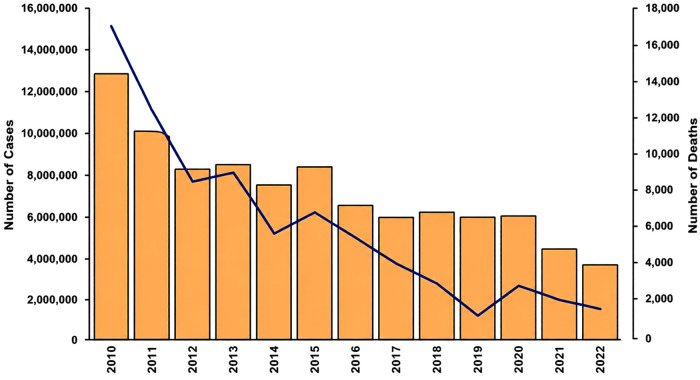
Trends and changes in malaria cases (y-axis) and deaths (alternative y-axis) between 2010 and 2022 in Tanzania.
