# Supplementary material for: The MARC SE-Africa dashboard: Joining forces to counteract emerging antimalarial resistance in South and East Africa
Source: PLOS Digit Health. 2026 May 6;5(5):e0000743. doi: 10.1371/journal.pdig.0000743 (PMC13148663; doi:10.1371/journal.pdig.0000743)
Supplement: S1 Table — (DOCX) [file pdig.0000743.s001.docx]

# S1 Table

# Summary of the prevalence and geographic distribution of the A578S *pfKelch13* genetic marker

| **Country** | **Site Name** | **Date Range** | **Prevalence range (%)** | **Ref.** |  |
| --- | --- | --- | --- | --- | --- |
| Angola | Huambo, Lunda Sul, and Zaire | 2015-2017 | 0.5-2.5 | (1, 2) |  |
| South Africa | Mpumalanga, pooled results from Mpumalanga, KwaZulu-Natal, and Limpopo provinces | 2022-2023 | 0.4-0.5 | Kawela M et al. manuscript in preparation |  |
| Uganda | Adjumani, Agago, Arua, Busia, Kwania, Kitgum, and Pader | 2016-2023 | 0.4-4 | (3-5) Asua et al. manuscript under review |  |
| Zambia | Pooled data from Southern and Western provinces | 2017 | 1.4 | Unpublished, Centre for International Health. University of Bergen. Norway_ University of Zambia |  |
| Kenya | Makamega, Western Province | 2015 | 3 | (6) |  |
| DRC | Mikalayi | 2017 | 0.9 | (6) |  |

# S1 Table references

ADDIN EN.REFLIST 1. Ménard D, Khim N, Beghain J, Adegnika AA, Shafiul-Alam M, Amodu O, et al. A worldwide map of Plasmodium falciparum K13-propeller polymorphisms. New England Journal of Medicine. 2016;374(25):2453-64.

2. Plucinski MM, Dimbu PR, Macaia AP, Ferreira CM, Samutondo C, Quivinja J, et al. Efficacy of artemether-lumefantrine, artesunate-amodiaquine, and dihydroartemisinin-piperaquine for treatment of uncomplicated Plasmodium falciparum malaria in Angola, 2015. Malar J. 2017;16(1):62.

3. Ogwang R, Osoti V, Wamae K, Ndwiga L, Muteru K, Ningwa A, et al. A retrospective analysis of P. falciparum drug resistance markers detects an early (2016/17) high prevalence of the k13 C469Y mutation in asymptomatic infections in Northern Uganda. Antimicrobial Agents and Chemotherapy. 2024;68(9):e01576-23.

4. Organization WH. World Health Organization Malaria Threats Map. 2024.

5. Angwe MK, Mwebaza N, Nsobya SL, Vudriko P, Dralabu S, Omali D, et al. Day 3 parasitemia and Plasmodium falciparum Kelch 13 mutations among uncomplicated malaria patients treated with artemether-lumefantrine in Adjumani district, Uganda. medRxiv. 2024.

6. WHO. WHO Malaria Threats Map 2024 [Available from: [https://apps.who.int/malaria/maps/threats/#/](#/).

7. Adam M, Nahzat S, Kakar Q, Assada M, Witkowski B, Tag Eldin Elshafie A, et al. Antimalarial drug efficacy and resistance in malaria-endemic countries in HANMAT-PIAM_net countries of the Eastern Mediterranean Region 2016-2020: Clinical and genetic studies. Trop Med Int Health. 2023;28(10):817-29.
