# Supplementary material for: The MARC SE-Africa dashboard: Joining forces to counteract emerging antimalarial resistance in South and East Africa
Source: PLOS Digit Health. 2026 May 6;5(5):e0000743. doi: 10.1371/journal.pdig.0000743 (PMC13148663; doi:10.1371/journal.pdig.0000743)
Supplement: S1 Text — (DOCX) [file pdig.0000743.s002.docx]

# S1 Text

# Titles and sources of published studies were used for data mining to determine the prevalence of pfKelch13 in South and East African countries.

- Continued Low Efficacy of Artemether-Lumefantrine in Angola in 2019
- Efficacy of artemether-lumefantrine. artesunate-amodiaquine. and dihydroartemisinin-piperaquine for treatment of uncomplicated Plasmodium falciparum malaria in Angola. 2015
- Historical trends and new surveillance of Plasmodium falciparum drug resistance markers in Angola
- MIM Conference. Kawela M et al Preliminary Regional Results from GenE8
- A Worldwide Map of Plasmodium falciparum K13-Propeller Polymorphisms
- Polymorphisms of Plasmodium falciparum k13-propeller gene among migrant workers returning to Henan Province. China from Africa
- Surveillance of Antimalarial Resistance Molecular Markers in Imported Plasmodium falciparum Malaria Cases in Anhui. China. 2012-2016
- Polymorphisms of pfcrt. pfmdr1. and K13-propeller genes in imported falciparum malaria isolates from Africa in Guizhou province. China
- Molecular surveillance of pfcrt. pfmdr1 and pfk13-propeller mutations in Plasmodium falciparum isolates imported from Africa to China
- Molecular Surveillance of Drug Resistance of Plasmodium falciparum Isolates Imported from Angola in Henan Province. China
- K13-propeller gene polymorphisms of Plasmodium falciparum and the therapeutic effect of artesunate among migrant workers returning to Guangxi. China (2014-2017)
- Molecular surveillance of Pfcrt and k13 propeller polymorphisms of imported Plasmodium falciparum cases to Zhejiang Province. China between 2016 and 2018
- Efficacy and safety of artemether-lumefantrine. artesunate-amodiaquine. and dihydroartemisinin-piperaquine for the treatment of uncomplicated Plasmodium falciparum malaria in three provinces in Angola. 2017
- Polymorphisms of Plasmodium falciparum k13-propeller gene among migrant workers returning to Henan Province. China from Africa.
- Molecular surveillance of Plasmodium falciparum drug resistance markers in clinical samples from Botswana.
- Unpublished, WHO Threats Map
- A Worldwide Map of Plasmodium falciparum K13-Propeller Polymorphisms.
- Identification of the PfK13 mutations R561H and P441L in the Democratic Republic of Congo
- Molecular surveillance of Kelch 13 polymorphisms in Plasmodium falciparum isolates from Kenya and Ethiopia.
- Novel Plasmodium falciparum k13 gene polymorphisms from Kisii County, Kenya during an era of artemisinin-based combination therapy deployment
- Resistance screening and trend analysis of imported falciparum malaria in NSW, Australia (2010 to 2016).
- Molecular surveillance of Pfcrt and k13 propeller polymorphisms of imported Plasmodium falciparum cases to Zhejiang Province. China between 2016 and 2018.
- Anti-malarial resistance in Mozambique: Absence of Plasmodium falciparum Kelch 13 (K13) propeller domain polymorphisms associated with resistance to artemisinins
- MIM Genomic surveillance of Plasmodium falciparum Drug Resistance Markers and Genetic Diversity in Mozambique
- MIM Conference. Kawela M et al Preliminary Regional Results from GenE8; 9th Annual Southern Africa Malaria Research Conference Conference 2024; Eloff, L, Genomic surveillance reveals artemisinin partial resistance markers in Northern Namibia in 2023 from 9th Annual Southern Africa Malaria Research Conference Conference 2024
- Association of Plasmodium falciparum kelch13 R561H genotypes with delayed parasite clearance in Rwanda: an open-label. single-arm. multicentre. therapeutic efficacy study.
- Escalating Plasmodium falciparum K13 marker prevalence indicative of artemisinin resistance in southern Rwanda
- Increase in Kelch 13 Polymorphisms in Plasmodium falciparum. Southern Rwanda.
- High Prevalence of Plasmodium falciparum K13 Mutations in Rwanda Is Associated With Slow Parasite Clearance After Treatment With Artemether-Lumefantrine.
- Emergence and clonal expansion of in vitro artemisinin-resistant Plasmodium falciparum kelch13 R561H mutant parasites in Rwanda.
- Expansion of artemisinin partial resistance mutations and lack of histidine rich protein-2 and -3 deletions in Plasmodium falciparum infections from Rukara, Rwanda
- "Malaria update: Increase in frequency of Kelch 13 mutations found
- in malaria parasites from Mpumalanga Province. an early warning of
- the possible emergence of artemisinin-resistant malaria"
- "Malaria treatment: first detection of the Plasmodium
- falciparum kelch 13 Q613E mutation in South Africa"
- Safety and tolerability of single low-dose primaquine in a low-intensity transmission area in South Africa: an open-label. randomized controlled trial.
- Absence of kelch13 artemisinin resistance markers but strong selection for lumefantrine-tolerance molecular markers following 18 years of artemisinin-based combination therapy use in Mpumalanga Province. South Africa (2001–2018).
- Making data map-worthy-enhancing routine malaria data to support surveillance and mapping of Plasmodium falciparum anti-malarial resistance in a pre-elimination sub-Saharan African setting: a molecular and spatiotemporal epidemiology study.
- Parasite clearance. cure rate. post-treatment prophylaxis and safety of standard 3-day versus an extended 6-day treatment of artemether-lumefantrine and a single low-dose primaquine for uncomplicated Plasmodium falciparum malaria in Bagamoyo district. Tanzania: a randomized controlled trial
- Describing the current status of Plasmodium falciparum population structure and drug resistance within mainland Tanzania using molecular inversion probes.
- Detection of mutations associated with artemisinin resistance at k13-propeller gene and a near complete return of chloroquine susceptible falciparum malaria in Southeast of Tanzania.
- Country wide surveillance reveals prevalent artemisinin partial resistance mutations with evidence for multiple origins and expansion of high level sulfadoxine-pyrimethamine resistance mutations in northwest Tanzania.
- Evidence of artemisinin partial resistance in northwestern Tanzania: clinical and molecular markers of resistance.
- Emerging threat of Partial Artemisinin ResistanceMarkers in P. falciparum Parasite Populations inmultiple geographical locations in highTransmission Regions of Uganda
- A retrospective analysis of P. falciparum drug resistance markers detects an early (2016/17) high prevalence of the k13 C469Y mutation in asymptomatic infections in Northern Uganda
- Day 3 parasitemia and Plasmodium falciparum Kelch 13 mutations among uncomplicated malaria patients treated with artemether-lumefantrine in Adjumani district. Uganda.
- Evolution of artemisinin partial resistance in Ugandan malaria parasites
- Changing Molecular Markers of Antimalarial Drug Sensitivity across Uganda.
- Changing Prevalence of Potential Mediators of Aminoquinoline. Antifolate. and Artemisinin Resistance Across Uganda.
- NMCP, Victor Asua
- Indigenous emergence and spread of kelch13 C469Y artemisinin-resistant Plasmodium falciparum in Uganda.
- Varied Prevalence of Antimalarial Drug Resistance Markers in Different Populations of Newly Arrived Refugees in Uganda
- Decreased susceptibility of Plasmodium falciparum to both dihydroartemisinin and lumefantrine in northern Uganda.
- Polymorphisms of Plasmodium falciparum k13-propeller gene among migrant workers returning to Henan Province, China from Africa.
- Efficacy of artesunate-amodiaquine and artemether-lumefantrine for uncomplicated Plasmodium falciparum malaria in Madagascar, 2018.
- Assessment of Plasmodium falciparum anti-malarial drug resistance markers in pfk13-propeller, pfcrt and pfmdr1 genes in isolates from treatment failure patients in Democratic Republic of Congo, 2018-2019.
