# Supplementary material for: The MARC SE-Africa dashboard: Joining forces to counteract emerging antimalarial resistance in South and East Africa
Source: PLOS Digit Health. 2026 May 6;5(5):e0000743. doi: 10.1371/journal.pdig.0000743 (PMC13148663; doi:10.1371/journal.pdig.0000743)
Supplement: S2 Table — (DOCX) [file pdig.0000743.s003.docx]

# S2 Table

# MARC SE-Africa countries reporting the highest malaria cases and deaths in relation to their estimated population adjusted (per 100,000).

| **Malaria cases** | | | | | |
| --- | --- | --- | --- | --- | --- |
| **Year** | **Country** | **Reported cases** | **Year** | **Country** | **Reported cases (population adjusted)** |
| 2010 | Tanzania | 12893899 | 2010 | Tanzania | 28,807.72 |
| 2011 | Tanzania | 10165442 | 2011 | Tanzania | 22,711.76 |
| 2012 | Tanzania | 8478109 | 2012 | Burundi | 23,107.8 |
| 2013 | Tanzania | 8587728 | 2013 | Burundi | 44,562.07 |
| 2014 | DRC | 10288519 | 2014 | Burundi | 50,406.09 |
| 2015 | DRC | 12538805 | 2015 | Burundi | 49,139.36 |
| 2016 | DRC | 16821130 | 2016 | Burundi | 79,593.68 |
| 2017 | DRC | 15060454 | 2017 | Burundi | 79,618.81 |
| 2018 | DRC | 15755949 | 2018 | Burundi | 44,955.65 |
| 2019 | DRC | 18228070 | 2019 | Burundi | 90,151.26 |
| 2020 | DRC | 18813663 | 2020 | Zambia | 42,610.04 |
| 2021 | DRC | 18005873 | 2021 | Burundi | 52,434.77 |
| 2022 | DRC | 22593991 | 2022 | Burundi | 60,936.69 |
| **Malaria deaths** | | | | | |
| **Year** | **Country** | **Reported deaths** | **Year** | **Country** | **Reported deaths (population adjusted)** |
| 2010 | Kenya | 26017 | 2010 | Kenya | 62.54302 |
| 2011 | DRC | 23748 | 2011 | Malawi | 45.01411 |
| 2012 | DRC | 21601 | 2012 | Malawi | 37.20375 |
| 2013 | DRC | 30918 | 2013 | DRC | 45.09427 |
| 2014 | DRC | 25502 | 2014 | DRC | 37.19497 |
| 2015 | DRC | 39054 | 2015 | DRC | 48.19367 |
| 2016 | DRC | 33997 | 2016 | Angola | 56.81197 |
| 2017 | DRC | 21320 | 2017 | Angola | 49.6026 |
| 2018 | Angola | 11814 | 2018 | Angola | 41.9564 |
| 2019 | DRC | 92232 | 2019 | DRC | 113.8167 |
| 2020 | Angola | 11757 | 2020 | Angola | 35.14679 |
| 2021 | Angola | 13676 | 2021 | Angola | 40.88352 |
| 2022 | Angola | 12474 | 2022 | South Sudan | 40.18627 |
