# Supplementary material for: The MARC SE-Africa dashboard: Joining forces to counteract emerging antimalarial resistance in South and East Africa
Source: PLOS Digit Health. 2026 May 6;5(5):e0000743. doi: 10.1371/journal.pdig.0000743 (PMC13148663; doi:10.1371/journal.pdig.0000743)
Supplement: S2 Text — (DOCX) [file pdig.0000743.s004.docx]

# S2 Text

# Kelch 13 genotyping

(("K13"[All Fields] OR "Kelch 13"[All Fields] OR "Pfk13"[All Fields] OR "kelch13"[All Fields] OR "Pfkelch13"[All Fields]) AND ("resistant*"[All Fields] OR "resistance"[All Fields]) AND ("malaria*"[All Fields] OR "antimalaria*"[All Fields]) AND ("artesunate*"[All Fields] OR artesunate SP*"[All Fields] OR "artemisinin*"[All Fields] OR "ACT"[All Fields]) AND ("Angola"[All Fields] OR "Botswana"[All Fields] OR "Burundi"[All Fields] OR "Comoros"[All Fields] OR "Democratic Republic of Congo"[All Fields] OR "Eswatini"[All Fields] OR "Kenya"[All Fields] OR "Madagascar"[All Fields] OR "Malawi"[All Fields] OR "Mozambique"[All Fields] OR "Namibia"[All Fields] OR "Rwanda"[All Fields] OR "Somalia"[All Fields] OR "South Africa"[All Fields] OR "South Sudan"[All Fields] OR "Tanzania"[All Fields] OR "Uganda"[All Fields] OR "Zambia"[All Fields] OR "Zanzibar"[All Fields])) AND ("delayed parasite clearance"[All Fields] OR "delayed clearance"[All Fields] OR "parasite clearance half-life"[All Fields]) AND ((7)[pdat])
