# Supplementary material for: The MARC SE-Africa dashboard: Joining forces to counteract emerging antimalarial resistance in South and East Africa
Source: PLOS Digit Health. 2026 May 6;5(5):e0000743. doi: 10.1371/journal.pdig.0000743 (PMC13148663; doi:10.1371/journal.pdig.0000743)
Supplement: S3 Table — (DOCX) [file pdig.0000743.s005.docx]

# S3 Table

# Links to additional resources for further information on resistance to antimalarial medications and resources to combat malaria

| Resource | Description | Link |
| --- | --- | --- |
| MARC SE-Africa | Malaria resistance profiles of South and East African countries | https://www.marcse-africa.org/ |
| WWARN | Analytic Tools | https://www.iddo.org/wwarn/tools-resources/analytical-tools |
| World Health Organization | The World Malaria Report | https://www.who.int/teams/global-malaria-programme/reports/world-malaria-report-2023 |
| The President's Malaria Initiatives | PMI country profiles | https://www.pmi.gov/country-profiles-2024/ |
| South African Medical Research Council | Malaria Research Group | https://www.samrc.ac.za/research/centre-and-platforms-office/malaria-research-group |
| The Roll Back Malaria Partnership to End Malaria | Dashboard | https://endmalaria.org/our-work-working-groups/case-management |
