# Supplementary material for: The MARC SE-Africa dashboard: Joining forces to counteract emerging antimalarial resistance in South and East Africa
Source: PLOS Digit Health. 2026 May 6;5(5):e0000743. doi: 10.1371/journal.pdig.0000743 (PMC13148663; doi:10.1371/journal.pdig.0000743)
Supplement: S3 Text — (DOCX) [file pdig.0000743.s006.docx]

# S3 Text

# WorldWide Antimalarial Research Network (WWARN) Clinical Trial Publication Library details

The WorldWide Antimalarial Research Network (WWARN) Clinical Trial Publication Library (WCTL; available at www.iddo.org/wwarn/wwarn-clinical-trials-publication-library) is a key resource for screening relevant literature in this study. The WCTL is maintained by the Infectious Diseases Data Observatory (IDDO), based at the University of Oxford. It provides curated literature on therapeutic efficacy study (TES) outcomes and *PfKelch13* genotyping. This extensive library compiles all antimalarial clinical efficacy trials published since 1946 and is updated biannually, ensuring comprehensive and current data.

**The following databases are searched for WCTL:**

MEDLINE, EMBASE, Web of Science (all Databases), Cochrane Central, WHO Global Index Medicus and Clinicaltrials.gov.

No restrictions are placed on language or publication date. Briefly, the search terms used in the strategy included “Malaria,” “malaria.ti,ab.”, “*Plasmodium*,” “plasmodium.ti,ab.”, “falciparum,” the names of each component of the antimalarial drug, and other related terms. A librarian provided a list of studies identified through the search results provided by the Bodleian Libraries librarian. These were uploaded into Covidence screening software and independently screened by two reviewers using the agreed inclusion and exclusion criteria. The final list of eligible studies is agreed upon, and two data extractors extract the study data into a REDCap database. The extracted data is available via the WWARN website to the broader malaria community.

The librarian performs literature searches through the Bodleian Library at Oxford University. The library's last update was on 31/08/2024; the next is scheduled for 28/02/2025.

The WCTPL includes randomised control trials, quasi-randomised controlled trials, case-control studies, and longitudinal cohort studies. Pharmacokinetic studies using drugs that are components of artemisinin-based combination therapy are also included. Animal studies, prevention studies, case reports, case series, systematic reviews, and literature reviews were excluded.

WCTL includes studies with at least 28 days of follow-up. Since the primary focus of this meta-analysis is parasite clearance evaluated in the first few days after starting antimalarial treatment, both studies within WCTPL and studies screened but excluded from WTPL were considered for inclusion. Two independent reviewers screened the title, abstract, and full text as necessary, and a third reviewer was used to resolve any discordances.
