# Supplementary material for: The MARC SE-Africa dashboard: Joining forces to counteract emerging antimalarial resistance in South and East Africa
Source: PLOS Digit Health. 2026 May 6;5(5):e0000743. doi: 10.1371/journal.pdig.0000743 (PMC13148663; doi:10.1371/journal.pdig.0000743)
Supplement: S4 Table — (DOCX) [file pdig.0000743.s007.docx]

# S4 Table

# Inclusion criteria and description of *pfKelch13* genotyping results

| **Criteria** | **Details** |
| --- | --- |
| **Genotyping details** | Genotyping results from samples of *P. falciparum* reporting on the *Kelch13* propeller gene region. |
| **Inclusion dates** | Data from samples collected between 2014 and the present. |
| **Types of literature** |  |
|  | - Case reports |
|  | - Cross-sectional studies |
|  | - Experimental, prospective, and observational studies |
|  | Studies reporting baseline or pretreatment infections. |
| **Conference data** | Conference abstracts or data shared directly by investigators. |
| **Genotyping information** | **Genotyping information must also have the following data available:** |
| - Baseline data | Genotyping results reported at baseline (Day 0). |
| - Country/Study site | Marker prevalence linked to a MARC SE-Africa country, preferably to a specific study site within the country. |
| - Data collection period | Marker prevalence associated with the year of data collection or a range not exceeding five years. |
| **Primary data** | Includes both experimental and observational studies on *pfKelch13* marker prevalence. |
