# Supplementary material for: The MARC SE-Africa dashboard: Joining forces to counteract emerging antimalarial resistance in South and East Africa
Source: PLOS Digit Health. 2026 May 6;5(5):e0000743. doi: 10.1371/journal.pdig.0000743 (PMC13148663; doi:10.1371/journal.pdig.0000743)
Supplement: S4 Text — (DOCX) [file pdig.0000743.s008.docx]

# S4 Text

# Scoping Review search terms

W included a scoping search was performed using PubMed with the following search terms: (PUBMED) (("K13"[All Fields] OR "Kelch 13"[All Fields] OR "Pfk13"[All Fields] OR "kelch13"[All Fields] OR "Pfkelch13"[All Fields]) AND "resistant*"[All Fields] AND ("malaria*"[All Fields] OR "antimalaria*"[All Fields])) AND ((7) [pdat]) AND ("artesunate*"[All Fields] OR "artesunate SP*"[All Fields]))

-Searches are performed monthly.
