# Supplementary material for: The MARC SE-Africa dashboard: Joining forces to counteract emerging antimalarial resistance in South and East Africa
Source: PLOS Digit Health. 2026 May 6;5(5):e0000743. doi: 10.1371/journal.pdig.0000743 (PMC13148663; doi:10.1371/journal.pdig.0000743)
Supplement: S5 Table — (DOCX) [file pdig.0000743.s009.docx]

# S5 Table

# Exclusion criteria for literature-based searches for *pfKelch13* genotyping results

| **Criteria** | **Details** |
| --- | --- |
| **Species focus** | Genotyping results from species other than *P. falciparum* were excluded. |
| **Geographic focus** | Genotyping results from countries outside the 19 MARC SE-Africa countries were excluded. |
| **Type of literature excluded** | Reviews, opinion pieces, letters to the editor, republished data, etc. |
| **Sample type** | Genotyping results from cultured strains or tissues other than blood or dried blood samples. |
| **Time frame exclusion** | Samples obtained outside the date range 2014-and the present. |
| **Geographic origin** | Samples with unknown geographic origin are excluded. |
| **Host species** | Samples obtained from non-human hosts. |
| **Gene region** | Genotype results other than the |
