# Supplementary material for: The MARC SE-Africa dashboard: Joining forces to counteract emerging antimalarial resistance in South and East Africa
Source: PLOS Digit Health. 2026 May 6;5(5):e0000743. doi: 10.1371/journal.pdig.0000743 (PMC13148663; doi:10.1371/journal.pdig.0000743)
Supplement: S6 Table — (DOCX) [file pdig.0000743.s010.docx]

# S6 Table

# *PfKelch13* marker prevalence data categories extracted per marker entry

| **Category** | **Description** |
| --- | --- |
| Country | The country where the samples for *pfKelch13* genotyping were collected. |
| District | The administrative region or district within the country where the sampling occurred. |
| Site name | The name of the location (e.g., clinic, village, or health facility) where the samples were collected. |
| Latitude | The geographical latitude coordinate of the sampling sit. |
| Longitude | The geographical longitude coordinate of the sampling site for accurate mapping. |
| Start year | The year when sample collection began at the specified site. |
| End year | The year when sample collection ended at the specified site. |
| Marker | The *pfKelch13* mutation (genotype) reported in the samples (e.g., C580Y, R539T). |
| Present | The total number of samples that had the genotype. |
| Tested | The total number of samples subjected to genotyping. |
| Prevalence (%) | The percentage of samples with the assigned genotype for the *pfKelch13* mutation out of the total tested. |
| PubMed ID/Unique Identifier | The unique identifier (e.g., PubMed ID or database reference number) for the study or data source. |
| Year published | The year in which the study or report providing the data was published. |
| Title | The title of the study or report that provided the genotyping data. |
| Authors | The authors of the study or report that contributed the data. |
| Publication URL/Data Reference URL | The URL link to the publication or data reference that reports on the genotyping results. |
