# Supplementary material for: The MARC SE-Africa dashboard: Joining forces to counteract emerging antimalarial resistance in South and East Africa
Source: PLOS Digit Health. 2026 May 6;5(5):e0000743. doi: 10.1371/journal.pdig.0000743 (PMC13148663; doi:10.1371/journal.pdig.0000743)
Supplement: S7 Table — (DOCX) [file pdig.0000743.s011.docx]

# S7 Table

# Inclusion and exclusion criteria for screening TES outcome studies

| **Inclusion Category** | **Description** | **Exclusion Category** | **Description** |
| --- | --- | --- | --- |
| **Results from MARC-SE-Africa countries** | Studies must involve data from one of the MARC SE-Africa countries. | **Non-human/animal infections** | Studies involving non-human or animal infections. |
| **Human infection** | The study must involve *Plasmodium falciparum* infections in humans. | **Non-WHO recommended antimalarials** | Studies using antimalarials not recommended by WHO. |
| ***Plasmodium* infections only** | Studies should focus on *Plasmodium* falciparum infections. | **Case reports, reviews, opinion pieces** | Non-primary research sources like reviews or case reports. |
| **WHO-recommended antimalarials** | Studies should evaluate WHO-recommended antimalarials (e.g., AL, DP, ASAQ, ASPY, PA). | **Intermittent screening** | Studies involving intermittent screening methods. |
| **Trial dates** | Trials conducted between 2014 and the current date. | **Asymptomatic infections** | Studies focusing on asymptomatic infections. |
| **PCR-corrected and uncorrected rates** | Studies must report PCR-corrected and uncorrected therapeutic efficacy rates. | **Healthy volunteers** | Studies involving healthy volunteers. |
| **Length of follow-up** | Follow-up periods of Day 28 and Day 42 are preferable. | **Experimental *or in vitro* investigations** | Laboratory-based or experimental studies. |
| **Day 3 parasite positivity (desired)** | Studies that report Day 3 parasite positivity rates are desirable. | **Prevention or prophylaxis studies** | Studies focused on prevention or prophylaxis. |
