# Supplementary material for: The MARC SE-Africa dashboard: Joining forces to counteract emerging antimalarial resistance in South and East Africa
Source: PLOS Digit Health. 2026 May 6;5(5):e0000743. doi: 10.1371/journal.pdig.0000743 (PMC13148663; doi:10.1371/journal.pdig.0000743)
Supplement: S8 Table — (DOCX) [file pdig.0000743.s012.docx]

# S8 Table

# WCTL literature search terms

| **Disease & Species Terms Included:** | | | | | | | | |
| --- | --- | --- | --- | --- | --- | --- | --- | --- |
| malaria | *falciparum* | | *vivax* | | *ovale* | | | *knowlesi* |
| *malariae* | *plasmodium* | |  | |  | | |  |
| **Drug Terms Included:** | | | | | | | | |
| ACT-451840 | | Amodiaquine | | Amopyroquin | | AQ-13 | Arteether | |
| Artefenomel | | Arteflene | | Artemether | | Artemether-Lumefantrine | Artemether-Lumefantrine-Amodiaquine | |
| Artemisinin | | Artemisinin-naphthoquine | | Arterolane | | Arterolane-piperaquine | Artesunate | |
| Artesunate-Amodiaquine | | Artesunate-Amodiaquine-Chlorpheniramine | | Artesunate-Mefloquine | | Artesunate-Piperaquine | Atoguanil | |
| Atovaquone | | Atovaquone-Proguanil | | Azithromycin | | Berberine | Bulaquine | |
| CDRI 97/78 | | Chloroquine | | Chlorpheniramine | | Chlorproguanil-dapsone | Chlorproguanil | |
| Cipargamin | | Clindamycin | | Cotrimoxazole | | Dapsone | Dihydroartemisinin- Piperaquine | |
| Dihydroartemisinin- Piperaquine-Trimethoprim | | Dihydroartemisinin | | Doxycycline | | Elubaquine | Erythromycin | |
| Ferroquine | | Fosmidomycin | | Fosmidomycin-piperaquine | | Ganaplacide | Ganaplacide-Lumefantrine | |
| GSK701 | | Halofantrine | | Imatinib | | INE963 | Ketotifen | |
| L9LS antibody | | Lotilaner | | Lumefantrine | | M5717 | M5717-pyronaridine | |
| MMV533 | | Mefloquine | | Metakelfin | | Methylene Blue | Methylene Blue-Amodiaquine | |
| Naphthoquine | | Norfloxacin | | P218 | | Pafuramidine | Pentaquine | |
| Piperaquine | | Primaquine | | Probenecid | | Proguanil | Pyrimethamine | |
| Pyronaridine | | Quinacrine | | Quinidine | | Quinine | Rifampicin-Cotrimoxazole-Isoniazid | |
| Rosiglitazone | | Ruxolitinib | | Sevuparin | | SJ733 | Spiramycin | |
| Sulfadoxine | | Sulfadoxine-Pyrimethamine | | Sulfadoxine-Pyrimethamine-Amodiaquine | | Sulfamethoxazole | Sulfamonomethoxine | |
| Sulfamethoxypyrazine | | Tafenoquine | | Tetracycline | | Trimethoprim | XTB-31F mAB | |
| ZY-19489 | | ZY-19489-ferroquine | |  | |  |  | |
