# Supplementary material for: The MARC SE-Africa dashboard: Joining forces to counteract emerging antimalarial resistance in South and East Africa
Source: PLOS Digit Health. 2026 May 6;5(5):e0000743. doi: 10.1371/journal.pdig.0000743 (PMC13148663; doi:10.1371/journal.pdig.0000743)
Supplement: S9 Table — (DOCX) [file pdig.0000743.s013.docx]

# S9 Table

# Data capture for TES outcome database

| **Category** | **Description** |
| --- | --- |
| Country | The MARC SE-Africa country where the clinical trial was conducted. |
| Region | The region (Eastern or Southern Africa) is assigned to the country according to the United Nations Geoscheme. |
| Start year | The year in which the clinical trial began. |
| End year | The year in which the clinical trial ended. |
| Site | The specific study site where the trial was conducted (e.g., health facility or district). |
| Latitude | The geographical latitude coordinate of the study site for mapping purposes. |
| Longitude | The geographical longitude coordinate of the study site for mapping purposes. |
| Antimalarial | The antimalarial or combination of drugs investigated in the trial (e.g., AL, DP, ASAQ, PA). |
| Follow up | The length of the participants' follow-up period is typically measured in days (e.g., Day 28, Day 42). |
| *P. falciparum-*arasite-positive | The number of participants remains positive for *Plasmodium falciparum* parasites at a given time (e.g., Day 3). |
| PCR Uncorrected ACPR | The uncorrected adequate clinical and parasitological response (ACPR) based on microscopy or clinical outcomes without PCR confirmation. |
| PCR-corrected | The PCR-corrected adequate clinical and parasitological response (ACPR), adjusting for reinfection or new infections. |
