# Supplementary material for: The MARC SE-Africa dashboard: Joining forces to counteract emerging antimalarial resistance in South and East Africa
Source: PLOS Digit Health. 2026 May 6;5(5):e0000743. doi: 10.1371/journal.pdig.0000743 (PMC13148663; doi:10.1371/journal.pdig.0000743)
Supplement: S10 Table — (DOCX) [file pdig.0000743.s014.docx]

# S10 Table

# Country partners, NMCPs, and focal point members per country

| Country | Name | Position |
| --- | --- | --- |
| Uganda | Jimmy Opigo | Program manager |
|  |  | EAC Focal point member |
| Rwanda | Dr. Aimable Mbituyumuremyi | Program manager/FP |
| Kenya | Kibor Kipkemoi Keitany | Program manager |
|  | Emmah Mongina Nyandigisi | EAC Focal point member |
| Burundi | Dr Pierre Sinarinzi | Program manager/FP |
| South Sudan | Peter Aguek Kon Baak | EAC Focal point member |
| DRC | Tommy Nseka Manbul | EAC Focal point member |
| Tanzania | Samwel Lazaro Nhiga | Program Manager |
|  | Sijenunu Aron Mwaikambo | EAC Focal point member |
| Zanzibar | Shija Joseph Shija | Program Manager |
|  | Ali Omar Hamdu | EAC Focal point member |
| Somalia | Abdikarin Hussein Hassan | EAC Focal point member |
| South Africa | Dr Jaishree Raman | National Reference Laboratory |
