# Supplementary material for: The MARC SE-Africa dashboard: Joining forces to counteract emerging antimalarial resistance in South and East Africa
Source: PLOS Digit Health. 2026 May 6;5(5):e0000743. doi: 10.1371/journal.pdig.0000743 (PMC13148663; doi:10.1371/journal.pdig.0000743)
Supplement: Attachment — Submitted filename: Response to Reviewer.docx [file pdig.0000743.s015.docx]

We thank the reviewers and the editor for their input and for providing the opportunity to improve our manuscript [PDIG-D-25-00012] - [EMID:a13d89b6f1a13ff7]. The reviewers’ comments are illustrated in bold, and the changes made to this manuscript are indicated using track changes. We have also noted minor changes made to improve the manuscript's overall readability.

**Reviewer 1:**

***Reviewer*: By offering interactive maps and charts, the dashboard allows stakeholders to monitor resistance patterns, assess policy relevance, and identify cross-border transmission risks. The tool has already supported national-level policy changes (e.g. Tanzania’s decision to switch first-line therapies).**

**The manuscript is clearly written, well-structured, and uses standard academic English. Occasional long paragraphs might benefit from editorial tightening for clarity and readability, but overall, the language is fluent, professional, and precise.**

*Response:* We thank the reviewer for their insight and kind words. Considerable effort has been made to edit and improve the manuscript's readability throughout. The changes are indicated in the attached manuscript.

***Reviewer*: Please discuss or address the following limitations:**

**- Data Gaps: Several countries (e.g., Eswatini, Botswana) lack recent TES or molecular data.**

**- Limited Analytical Depth: Primarily descriptive; lacks predictivel analytics.**

**- Dependence on Connectivity: Dashboard requires stable internet access, which may hinder use in remote areas. Is an offline version possible?**

**- Surveillance Quality Variability: Relies on external data sources of varying completeness and accuracy.**

**- Pfkelch13 Focus: While relevant, Pfkelch13 is not the only genetic marker of resistance—dashboard may miss broader resistance trends.**

*Response:*

1. Data gaps (Eswatini, Botswana, etc.). We agree that the absence of recent TES and molecular data from several countries, including Eswatini and Botswana, is a limitation hampering insight into emerging antimalarial resistance in southern Africa and neighbouring countries. The dashboard can only display data generated and made accessible by national programmes and their partners. At the same time, one of the main strengths of a collated regional dataset is that it makes evidence gaps visible, thereby making it easier for ministries, regional bodies, and funders to prioritise where data collection and expedited evidence sharing are most needed. The dashboard's strength lies in collating all available information to provide regional-level insight. This is particularly relevant in low transmission settings, where obtaining sufficient numbers of infections for adequately powered TES or molecular surveys is more challenging. Through MARC SE-Africa, we are already using the dashboard outputs to inform conversations with countries and initiatives such as GenE8, SADC, and the EAC, on where new studies and enhanced surveillance are most urgently needed.

To address the reviewers' concerns, we have included the following in the discussion section of this manuscript:

Lines 541-546: “Importantly, by displaying data at a regional scale, the dashboard also reveals where TES and molecular surveillance are absent or outdated, including in pre-elimination settings such as Eswatini and Botswana. These visible gaps can support national programmes, regional bodies, and donors to prioritise data collection and expedited evidence sharing, while recognising that in low transmission settings the small number of infections often limits the feasibility of adequately powered TES and molecular surveys.”

2. Descriptive and predictive insight

We agree that the analyses presented in this manuscript are primarily descriptive. This is intentional. The main objective here is to introduce the MARC SE-Africa dashboard, describe how the underlying dataset was curated, and present the core descriptive insights most relevant to policy. Building predictive models on top of this harmonised dataset is an essential next step. This work is already underway in collaboration with the WorldWide Antimalarial Resistance Network (WWARN) and Infectious Diseases Data Observatory (IDDO), where the MARC SE-Africa dataset and dashboard insights are being used for risk mapping and predictive modelling of antimalarial resistance. Early results from this work were presented at the American Society of Tropical Medicine and Hygiene (ASTMH) annual meeting in 2025 (Symposium 70: “Antimalarial drug resistance in Africa – Mapping the spread of drug resistance in Africa”, presentation by Dr Lucy Harrison, Oxford University). A separate manuscript dedicated to these modelling analyses is in preparation, and when available, the MARC SE-Africa dashboard will offer a link to these predictive maps.

3. Dashboard dependency on internet connectivity

We acknowledge that the dashboard’s web-based nature requires internet connectivity, which can be intermittent in some endemic areas. Most interactive public health dashboards share this limitation. To partially mitigate this, we have ensured that all collated datasets and the associated code are openly available for download (via the AntimalRepo repository), allowing users to analyse the data offline, generate static figures, and produce country-specific reports even when connectivity is limited. National Malaria Programmes and their partners have already used exported images and tables in briefing documents and presentations for settings with unreliable internet access.

We also note that mobile broadband coverage has expanded rapidly in sub-Saharan Africa, with recent GSMA reports indicating that only about 10%-13% of the population now lives outside mobile broadband coverage. (https://www.gsma.com/solutions-and-impact/connectivity-for-good/mobile-for-development/blog/mobile-connectivity-in-sub-saharan-africa-4g-and-3g-connections-overtake-2g-for-the-first-time). This expansion has occurred alongside a steady rise in smartphone adoption across Africa, meaning that an increasing proportion of users can access web-based tools directly from their mobile devices. As a result, the interactive dashboard can also be viewed and used on a smartphone, further improving accessibility in resource-limited settings.

This suggests that connectivity will increasingly support the use of web-based tools, even though affordability and digital skills remain barriers. Developing a fully offline version of the interactive dashboard would require substantial additional development and maintenance resources beyond the scope of the current grant. Still, the open data and code already enable offline use for many key functions.

4. Surveillance quality

We agree that the dashboard can only be as reliable as the surveillance systems and reporting mechanisms on which it draws. We explicitly recognise this in the Discussion by stating that the accuracy of epidemiological indicators depends on the strength of national health information and other surveillance systems and that inconsistencies in reported malaria cases and deaths are a concern in several countries.

To improve data quality, we did not rely on a single source. Instead, we triangulated across the World Malaria Report, national malaria treatment guidelines, MARC SE-Africa country profiles, President’s Malaria Initiative country reports, and WHO threat maps, and other published and peer-reviewed sources. Then we sought review and correction from the relevant NMP. Discrepancies between WHO and country data were documented transparently in the GitHub repository for this dataset.

5. Insights beyond PfKelch13

We agree that Pfkelch13 is only one part of the resistance landscape. The current focus on Pfkelch13 reflects both the MARC SE-Africa grant mandate, which is centred on artemisinin partial resistance, and the current WHO emphasis on Pfkelch13 as the primary molecular marker for surveillance of delayed parasite clearance. At the same time, partner drug resistance (for example, markers in pfcrt, pfmdr1, and plasmepsin genes) is critical for understanding the full efficacy of ACT, as is diagnostic resistance (such as Pfhrp2/3 deletions).

To address this, we designed the dashboard to sit alongside, rather than replace, complementary molecular resources. The manuscript already notes that the dashboard links to WWARN tools and resources, including the Molecular Surveyor and WHO Malaria Threats Maps. To address the reviewers' concerns, we have added the following lines to the manuscript:

Lines 320-325: “The focus on *Pfkelch13* therefore provides a detailed view of ART-R, while links to external tools such as the WWARN Molecular Surveyor and WHO Malaria Threats Maps allow users to examine partner drug and diagnostic resistance markers in parallel, so that the dashboard complements rather than replaces broader molecular surveillance resources.”

***Reviewer*: Evaluation of data: The authors should discuss confounding variables. Moreover, although trends are reported, causality or prediction is not explored.**

*Response*: We agree that the present work describes trends and patterns but does not aim to establish causality or build formal prediction models. This manuscript is intended as a methods and descriptive paper that introduces the dashboard, documents the data curation process, and presents key descriptive insights most relevant to policy discussion. We have made this scope more explicit in the Discussion and clarified that predictive modelling and more formal causal analysis are the subject of ongoing work with WWARN and IDDO, using the MARC SE Africa dataset. These studies will apply appropriate multivariable and spatiotemporal methods to explore associations between molecular markers, treatment policies, and clinical outcomes. Moreover, the dashboard presents current trends where available, but there are indeed other potential contributors to the emergence, selection and spread of resistance. These include differences in transmission intensity, age structure and immunity of the treated population, malaria treatment policy and practice – including in the private and informal sectors, access to care, diagnostic practices, study design, and the quality and completeness of resistance surveillance data. To address the reviewer’s concerns, we have included the following sentence in the discussion section of this manuscript.

Lines 556-561: “The dashboard and analyses presented here are descriptive in nature and were not designed to adjust fully for confounding or to make causal or predictive inferences, which will require separate, dedicated modelling studies building on this curated dataset”.

***Reviewer*: Recommendation for Authors: include literature/data and please discuss:**

**- Regional coordination challenges; The dashboard’s policy impact; Technical limitations of Pfkelch13-only surveillance**

**e.g. in detail:**

**1) Recent Advances in ART-R Research in Africa**

**2) Digital Dashboard Evaluation and Use in general**

**3) Cross-Border Surveillance & Migration Impact**

**4) Genomic Surveillance Techniques & Broader Resistance Markers (especially for Plasmodium falciparum)**

*Response:* We thank the reviewer for their insight and share their sentiments listed above. We would, however, like to note that many of these aspects are already addressed in the current manuscript, and we are cautious about substantially expanding an already lengthy paper.

1. The Introduction summarises recent work on artemisinin partial resistance and remaining knowledge gaps across Africa, citing Rosenthal et al. 2024 (Lancet Infect Dis), Asua et al. 2021 (J Infect Dis), and related studies (refs 1, 3, 4, 6, 9, 10, 16, 17, 22–26, 43, 44).

The Methods further detail the genomic surveillance landscape by listing key resources used to generate Pfkelch13 prevalence data, including the WWARN Clinical Trial Library, WWARN Molecular Surveyor, and the WHO Malaria Threats Map (Table 2, refs 2, 27–31).

We also explicitly acknowledge that Pfkelch13 alone is insufficient and that other markers are essential for understanding partner-drug resistance in the Discussion.

2. We discuss the broader role of digital dashboards in infectious-disease management, including their use during COVID-19, with supporting literature on dashboard evaluation and public-health decision-making (refs 65–69). We agree that documenting how the dashboard is used in practice is important. In the revised Discussion, we now make it clearer that the MARC SE-Africa dashboard has already served as a key source of information for regional networks in East and Southern Africa. The collated datasets and country profiles have contributed to the Regional Detailed Action Plan for Responding to Antimalarial Drug Resistance in East Africa, which was endorsed by East African Community (EAC) ministers of health in May 2025, and to a draft antimalarial resistance profile and regional pathway that is currently under review within the Southern African Development Community (SADC). For the first time in decades, SADC will include a chapter on drug-resistant malaria, based in part on these data, in its annual SADC malaria report, to be tabled at the SADC Ministers of Health meeting in January 2026.

We also mention in our response here that the information displayed on the dashboard has been used by partners such as Maisha Meds in further analyses and webinars examining coordination challenges among the pharmaceutical sector, regulatory bodies, and other antimicrobial resistance stakeholders, and the implications of these gaps for the global response. Given the already substantial length of the manuscript, we have limited our revision to a brief description of these policy uses and direct interested readers to the regional reports and webinar materials for more detail:

Lines 461-468: “the dashboard has already informed regional planning processes: its collated datasets and country profiles contributed to the East African Community Regional Detailed Action Plan for Responding to Antimalarial Drug Resistance, endorsed by the region’s ministers of health in 2025, and to a draft regional antimalarial resistance profile and action pathway for the Southern African Development Community. In addition, partners such as Maisha Meds have drawn on the dashboard outputs in analyses and webinars that explore coordination challenges between pharmaceutical, regulatory and other antimicrobial resistance stakeholders.”.

3. The Methods and Discussion both acknowledge that the dashboard focuses on Pfkelch13 because of the MARC SE-Africa mandate and current WHO guidance, but that additional markers (e.g. *pfcrt, pfmdr1, pfplasmempsin* and others) are critical to fully understanding drug-resistant malaria. Table 3 explicitly includes “Other” Pfkelch13 variants to flag potentially important signals beyond the validated and candidate markers. In the Discussion, we further state that Pfkelch13 markers alone do not provide sufficient evidence on drug-resistant malaria and that broader molecular surveillance is needed, referencing WWARN tools and related literature (please see the response above) and the addition of the following lines to the manuscript:

Lines 320-325: “The focus on *Pfkelch13* therefore provides a detailed view of ART-R, while links to external tools such as the WWARN Molecular Surveyor and WHO Malaria Threats Maps allow users to examine partner drug and diagnostic resistance markers in parallel, so that the dashboard complements rather than replaces broader molecular surveillance resources.”

***Reviewer*: Please add future directions and goals for the dashboards presented. Is the integration of predictive analytics (machine learning a possible development? What is needed to integrate it in your dashboard?).**

*Response*: We thank the reviewer for this suggestion and agree that predictive analytics and machine-learning approaches represent an important next step for antimalarial resistance surveillance. In the present manuscript, however, our primary aim is to introduce the MARC SE-Africa dashboard, document the curation and structure of the underlying dataset, and present the core descriptive patterns most immediately valuable for national programmes and regional partners. Please note our response above regarding predictive modelling.

As noted in the Discussion, we already indicated that this work serves as a descriptive platform for more complex analytical studies, including ongoing modelling in collaboration with WWARN/IDDO, which will be reported in separate manuscripts. When available, the MARC SE-Africa dashboard will offer a link to these predictive maps.

**Reviewer: General Observations on figures: The figures are data-rich, policy-relevant, and well-integrated into the manuscript narrative. Most figures rely on aggregated descriptive data — predictive modeling overlays could be a next step.**

*Response*: We agree that expanding genomic surveillance beyond Pfkelch13 is scientifically essential and ultimately necessary for a comprehensive view of antimalarial drug resistance. As noted in our earlier responses on the technical limitations of *Pfkelch13*-only surveillance, the current dashboard focus reflects the specific scope of the MARC SE-Africa grant, which centres on artemisinin partial resistance, and current WHO guidance that prioritises *Pfkelch13* as the primary molecular marker for this phenotype. In the manuscript, we already acknowledge that *Pfkelch13* alone is insufficient to capture partner-drug resistance, and we direct readers’ attention to complementary resources such as the WWARN Molecular Surveyor and the WHO Malaria Threat Maps, which provide broader coverage of markers in genes such as pfcrt, pfmdr1, and others.

**Reviewer: Please discuss if expanding genomic surveillance beyond Pfkelch13 is possible?**

*Response:* We appreciate this suggestion and agree that, in principle, offline-compatible or lightweight mobile versions could further extend access. As noted above, however, the current MARC SE-Africa dashboard was designed and funded as a web-based, richly interactive tool that displays a wide range of indicators, maps, and stratifications on a single page. A substantial simplification into a lightweight app would risk losing important nuance in how the data are presented, and developing and maintaining parallel offline or mobile-native versions would fall beyond the scope and deliverables agreed with our current funders. In practice, the existing web dashboard is already being used by national and regional partners, who often export figures and tables for offline use. We therefore acknowledge this as a potential future direction, but respectfully consider it outside the remit of the present project and manuscript.

**Reviewer: Third, developing offline-compatible versions or lightweight mobile apps could expand access in regions with limited internet infrastructure. Is this an option? Please discuss ths use of the dashboards here.**

*Response:* We thank the reviewer for these encouraging comments and fully share their view of the dashboard's potential to support efforts to reduce malaria morbidity and mortality. In the Introduction, we already describe how sub-Saharan Africa accounts for 94% of global malaria cases and 95% of deaths, and how emerging artemisinin partial resistance threatens to substantially increase malaria-related morbidity and mortality in the region, contrasting this with the earlier emergence of artemisinin resistance in Southeast Asia.

We also outline how the dashboard was specifically designed to address these challenges by collating up-to-date data on *Pfkelch13* markers, ACT efficacy, national treatment policies, and malaria cases and deaths at regional, national, cross-border, and sub-national levels, to inform more targeted and coordinated responses within African-specific settings. These were the parameters that NMPs were most important for easy “one-stop” access. Such expedited data sharing is a first step to inform evidence-based responses to the threats posed by drug-resistant malaria. The Results and Data accessibility sections further emphasise that the dashboard links spatiotemporal trends in resistance to case and death metrics and provides direct access to WHO World Malaria Reports, the Malaria Threats Map, RBM dashboards, PMI/MARC SE-Africa country reports, and WWARN tools, with the explicit aim of supporting programme decisions that can prevent increases in deaths and illness.

The broader comparison between Africa and Asia regarding artemisinin resistance and its implications is covered through the cited literature on the emergence and dynamics of artemisinin partial resistance in both regions (e.g., Rosenthal et al., White & Chotivanich, WHO strategy documents, and Southeast Asian clinical studies).

Given the already substantial length of the manuscript, we respectfully propose not to expand this further in the text. Still, we believe the current Introduction, Results, and linked references already provide the requested context on how the dashboard contributes to efforts to reduce malaria burden in Africa and how the region’s situation relates to that of Asia.
